# Clinical trials with mechanism evaluation of intervention(s): mind the power and sample size calculation

**DOI:** 10.1186/s13063-024-08358-5

**Published:** 2024-08-06

**Authors:** Kim May Lee, Jennifer Hellier, Richard Emsley

**Affiliations:** Department of Biostatistics and Health Informatics, Institute of Psychiatry, Psychology and Neuroscience, King’s College London, 16 De Crespigny Park, London, SE5 8AF UK

**Keywords:** Mechanism evaluation, Mediation analysis, Power, Randomized controlled trials, Sample size calculation

## Abstract

**Background:**

Mediation analysis, often completed as secondary analysis to estimating the main treatment effect, investigates situations where an exposure may affect an outcome both directly and indirectly through intervening mediator variables. Although there has been much research on power in mediation analyses, most of this has focused on the power to detect indirect effects. Little consideration has been given to the extent to which the strength of the mediation pathways, i.e., the intervention-mediator path and the mediator-outcome path respectively, may affect the power to detect the total effect, which would correspond to the intention-to-treat effect in a randomized trial.

**Methods:**

We conduct a simulation study to evaluate the relation between the mediation pathways and the power of testing the total treatment effect, i.e., the intention-to-treat effect. Consider a sample size that is computed based on the usual formula for testing the total effect in a two-arm trial. We generate data for a continuous mediator and a normal outcome using the conventional mediation models. We estimate the total effect using simple linear regression and evaluate the power of a two-sided test. We explore multiple data generating scenarios by varying the magnitude of the mediation paths whilst keeping the total effect constant.

**Results:**

Simulations show the estimated total effect is unbiased across the considered scenarios as expected, but the mean of its standard error increases with the magnitude of the mediator-outcome path and the variability in the residual error of the mediator, respectively. Consequently, this affects the power of testing the total effect, which is always lower than planned when the mediator-outcome path is non-trivial and a naive sample size was employed. Analytical explanation confirms that the intervention-mediator path does not affect the power of testing the total effect but the mediator-outcome path. The usual effect size consideration can be adjusted to account for the magnitude of the mediator-outcome path and its residual error.

**Conclusions:**

The sample size calculation for studies with efficacy and mechanism evaluation should account for the mediator-outcome association or risk the power to detect the total effect/intention-to-treat effect being lower than planned.

## Introduction

The analysis of well-designed randomized controlled trials can be used for more than estimating an average treatment effect that represents the total effect of an intervention on outcome. For example, trials supported by UK’s National Institute of Health and Social Care (NIHR) Efficacy and Mechanism Evaluation program are designed to provide evidence about the underlying causal mechanisms that result in treatment induced change in clinical outcomes [1]. The US National Institute of Mental Health (NIMH) had also released an experimental medicine initiative [2] that all clinical trials had to demonstrate a target mechanism [3].

Mediation analysis is one of the statistical tools for gaining insight into the mechanisms of treatment effects on outcomes. It is typically used to investigate the role of an intermediate outcome *M* as a mediator of the relationship between intervention *X* and clinical outcome *Y*. This study of mediation of treatment effects, or how and why an intervention works, can deliver improved understanding of interventions and how these should be implemented in routine care [4, 5].

More specifically, the mediation model aims to decompose the total treatment effects into an indirect effect through a mediator variable (the effect of *X* on *Y* due to *M*) and the direct effect (the effect of *X* on *Y* controlling for *M*). The direct effect includes any causal mechanism not operating through the mediator(s) of interest. Furthermore, the total effect of *X* on *Y* equals the sum of the indirect and direct effects under some assumptions [6], which corresponds to the traditional intention-to-treat estimate.

Statistical methods for estimating and testing direct and indirect effects are well-developed [7–9]. They can be achieved via several methods including regression-based tests, structural equation modeling [10], and bootstrapping. Bootstrap or resample methods have been shown to be preferable to the joint-significance method because they can provide asymmetric confidence intervals [11, 12]. Furthermore, it has been shown that the power of testing the indirect/mediated effect can be higher than the power of testing the total effect or a direct effect under some parameter configurations [13, 14], for example, when the magnitudes of indirect effect and total effect are the same under complete mediation [10, 15, 16].

Nevertheless, mediation analyses are often secondary to the primary analysis of the total treatment effect [17]. It is implemented to understand the mechanisms by which an exposure affects an outcome through a mediating variable [6, 7]. As part of the initial study design, mediators are selected on the basis of theory and prior research. Yet, the sample size calculation of the study often focuses only on the characteristics of the primary outcome. Information about the mediation pathways is not typically accounted for in the calculation of power/sample size for the primary analysis [14].

The aim of this paper is to study the extent to which the strength of the mediation pathways, i.e., the intervention-mediator path and the mediator-outcome path respectively, may affect the power to detect the total effect, which would correspond to the intention-to-treat effect in a randomized trial. We conduct a simulation study to evaluate this in the context of a two-arm trial with a continuous mediator and a normal outcome. To our knowledge, this has not been explored by researchers in the field of mediation analysis and clinical trials.

In the next section, we describe the set-up of our simulation study. We provide an analytical explanation to the simulation finding and propose to account for the magnitude of the mediator-outcome path and its residual error in the consideration of effect size for sample size computation. We discuss the limitations of our investigation and make suggestions to conclude our work.

## Method

Consider a two-arm trial setting with a continuous outcome *Y* and a continuous mediator *M*. We want to examine the relation between the model parameters of mediation analysis and the power of the test of total effect. The set-up of our investigation is as follows.

As in the usual two-arm trial setting, we consider the sample size per arm according to the following formula for a two-tailed test:

$$\begin{aligned} n= 2 \left( \frac{Z_{1-\alpha /2} + Z_{1-\beta } }{\delta } \right) ^2 \end{aligned}$$

where $Z_{z}$ is the critical value of the normal distribution at *z* value, $\alpha$ and $\beta$ are the type one and type two error rate respectively, and $\delta$ is the standardized effect size under the alternative hypothesis.

For illustration purpose, we consider a simulation study for a two-arm study that aims to have 80% power to detect $\delta =0.5$ at $\alpha =0.05$ significance level. The required sample size per arm is $n=63$ without accounting for the presence of missing outcome.

Let *X* be the randomization variable that takes value of 1 if a patient is randomized to the experimental arm and 0 if to the control arm. Without loss of generality, we set 63 subjects to have $X=0$ and 63 subjects to have $X=1$ instead of adding a layer of variability in our simulation from using a randomization procedure. We simulate mediator, *M*, and outcome data, *Y*, respectively, according to the following models, which is commonly considered in a simple mediation analysis:

1 $$\begin{aligned} M & = i_m + a X + \epsilon _m \end{aligned}$$

2 $$\begin{aligned} Y & = i_y + c^{\prime } X + b M + \epsilon _y \end{aligned}$$

The parameters $i_m$ and $i_y$ are the model intercepts, parameter *a* describes the relation between the randomization variable *X* and the mediator *M*, parameter *b* describes the relation between the *M* and *Y* adjusting for *X*, and parameter $c^{\prime }$ describes the relation between *X* and *Y* adjusting for *M*. The error terms $\epsilon _m$ and $\epsilon _y$ reflect the variability in *M* that is not explained by *X* and the variability in *Y* that is not explained by its relations with *X* and *M*, respectively.

Mathematically, an indirect effect (i.e., the effect of *X* on *Y* due to *M*) is defined as the product of coefficients *a* and *b*, while $c^{\prime}$ is known as a direct effect of *X* on *Y* that is not mediated through *M*. The total effect can be defined as the sum of the indirect effect and the direct effect under some assumptions [6], i.e., $a b + c^{\prime }$.

In our simulation, we set $i_m=0.4, i_y=-0.4$; these values will not affect the finding. We simulate the error terms $\epsilon _y \sim N(0, \sigma ^2_y)$ with $\sigma ^2_y=1$ as we assume a standardized treatment effect and $\epsilon _m \sim N(0, \sigma ^2_m)$ with $\sigma ^2_m=\{0.5, 1\}$ for the investigation of the power of testing total effect. We consider scenarios with varying values of $a, b, c^{\prime}$, where the total effect is kept at 0.5, with $b=\{0, 0.1, 0.2, 0.3, 0.4\}$, $c^{\prime }=\{0, 0.1, 0.2, 0.3, 0.4, 0.5\}$, and $a=\{0, (0.5-c^{\prime }/b)\}$ and some scenarios with a null total effect where $a=c^{\prime }=0$ and $b=\{0.1, 0.2, 0.3, 0.4\}$. The way we simulate the data is such that the difference between the two dataset with different $\sigma ^2_m$ but same $a, b, c^{\prime }$ is in the values of the mediator.

For each simulated dataset, we fit the following simple linear regression model,

3 $$\begin{aligned} Y= i + c X + \epsilon \end{aligned}$$

and test the null hypothesis, $H_0: c = 0$ against alternative, $H_A: c \ne 0$ at 5% significance level. For each combination of $\sigma ^2_m, a, b, c^{\prime }$, we repeat the data generating step and the testing step 100,000 times to compute the frequency of rejecting $H_0$. This frequency is the type one error rate of the test of the total effect for scenarios with a null effect; it is the power of the test for all other scenarios where there is a direct effect or indirect effect or both. The maximum margin error is $1.96 \sqrt{0.5(1-0.5)/100000}=0.0031$ in our simulation. All simulations are conducted on R 4.2.2.

### Simulation results

The empirical type one error rate for scenarios with $a=c^{\prime }=0$ and $b=\{0.1, 0.2, 0.3, 0.4\}$ is $\{0.0538, 0.0532, 0.0528, 0.0524\}$ when $\sigma ^2_m=0.5$ and $\{0.0537, 0.0532, 0.0532, 0.0525\}$ when $\sigma ^2_m=0.5$ respectively. These are close to the nominal value of 0.05 plus the maximum margin error.

**Fig. 1:**
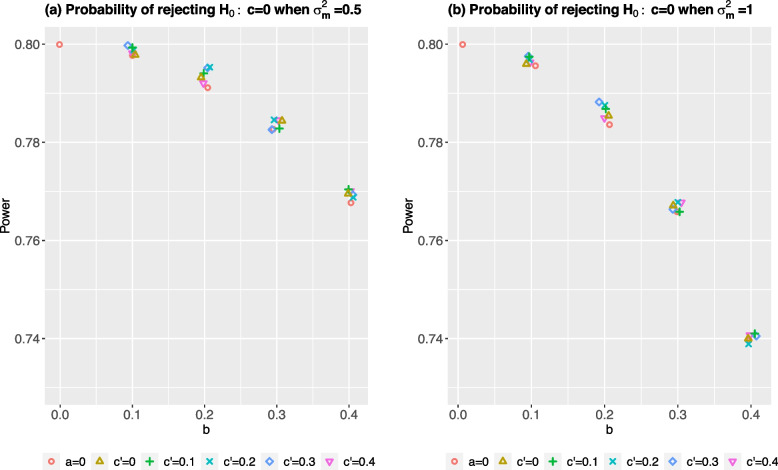
Empirical power of rejecting the null hypothesis following a simple linear regression analysis. The data generating mechanisms in the scenarios of plot (**a**) has $\sigma ^2_m=0.5$, and plot (**b**) $\sigma ^2_m=1$. All scenarios have $\sigma ^2_y=1$, total effect $c=0.5$, and $n=63$. The larger variability observed between the power for *b *= 0.2 is due to the Monte Carlo simulation error

Figure 1a and b show the power of the test of the total effect following the simple linear regression when the underlying data generating mechanism has $\sigma ^2_m=0.5$ and $\sigma ^2_m=1$, respectively. When $a=b=0$ and $c^{\prime }=0.5$, the power of 80% is obtained for the scenarios with $\sigma ^2_m=\{0.5, 1\}$ as expected, as the direct effect is equivalent to the total effect in the absence of an indirect effect, *ab*. When there is an indirect effect, i.e., $a\ne 0$ and $b\ne 0$, comparing the power for the scenarios with the same combination of $a, b, c^{\prime }$ but different $\sigma ^2_m$, we see that the power of the test of the total effect is higher when $\sigma ^2_m=0.5$ than when $\sigma ^2_m=1$. Moreover, the power of most cases are below 80% when there is an indirect effect.

Within each plot, for scenarios with the same $b>0$, we see that varying *a* and $c^{\prime }$ have little impact on the power, but this power is less than 80% even though the true total effect is 0.5. We also see that the power for scenarios with different *b* decreases with the magnitude of *b*. These observations are consistent across the scenarios with $\sigma ^2_m=\{0.5, 1\}$ .

### Analytical explanation

At first glance, the observations about the power when there is an indirect effect are unanticipated as the sample size of the design was computed to detect a total effect size of 0.5 with a power of 80%, and $\sigma ^2_y=1$ in the simulation. Upon inspecting the estimated total effect and its standard error across the simulated replications, we find that the estimate of total effect from the simple linear model (3) is unbiased across the considered scenarios, but the mean of its standard error increases with the magnitude of *b* (the magnitude of increase is larger when $\sigma ^2_m=1$ than when $\sigma ^2_m=0.5$ for the scenarios with the same combination of $a, b, c^{\prime }$). In other words, the standard error of the estimated *c* in model (3) varies with the magnitude of *b* and $\sigma ^2_m$.

Why is that the case? When we regress *Y* on *X*, the total variability in *Y* is decomposed into the variability explained by the treatment group *X* (i.e., experimentation error) and a nuisance source of variation in the outcome. Recall that the outcome is generated from models (1) and (2). Under such a data generating mechanism, the nuisance source of variation consists of the variability in the mediator and the residual error. Mathematically, we can substitute Eq. (1) into (2), resulting in

$$\begin{aligned} Y & = i_y + c^{\prime } X + b (i_m + a X + \epsilon _m ) + \epsilon _y \\ & = i + (c^{\prime } + a b) X + \epsilon \\ & = i + c X + \epsilon \end{aligned}$$

where $i^{\prime }=i_y+ b i_m$ is an intercept and $\epsilon = b \epsilon _m + \epsilon _y$ is the variability in the outcome not explained by *X*, i.e., the aforementioned nuisance source of variation in the simple linear regression model. It is obvious that $\epsilon$ has zero mean and a variance of $\sigma ^2=b^2 \sigma ^2_m + \sigma ^2_y$, which is different to the commonly used mathematical form of variance in the simple regression model.

With backward calculation and given a total sample size, we can compute the power to detect an effect size of

4 $$\begin{aligned} \delta ^{\prime }= \frac{\mu / \sigma _y }{ \sqrt{ b^2 \sigma ^2_m + \sigma ^2_y} } \end{aligned}$$

when the outcome data follows the mediation analysis models, where $\mu$ is the unstandardized effect size as considered in the usual sample size calculation. For $\delta = \mu / \sigma _y$ = 0.5, $\sigma ^2_y=1$, and varying values of *b* and $\sigma ^2_m$, Table 1 shows the true standardized effect size and the analytical power for the scenarios that we considered in the simulation. Comparing the corresponding power to the one in Fig. 1, the empirical power from the simulation is closed to the analytical power. This confirms that when the true data generating mechanism follows the mediation analysis models, the power (or equivalently the required sample size) depends on the magnitude of *b* path and the variability in the residual error of the mediator, i.e., $\sigma ^2_m$, but not the intervention-mediator path.

**Table 1:** Power to detect an effect size of $\delta ^{\prime }=\frac{0.5}{\sqrt{b^2 \sigma ^2_m + 1}}$ given the sample size per arm, $n=63$

		*b *= 0	*b *= 0.1	*b *= 0.2	*b *= 0.3	*b *= 0.4
$\sigma ^2_m = 0.5,$	$\delta ^{\prime }$	0.500	0.499	0.495	0.489	0.481
	Power	0.801	0.799	0.794	0.784	0.770
$\sigma ^2_m = 1,$	$\delta ^{\prime }$	0.500	0.498	0.490	0.479	0.464
	Power	0.801	0.797	0.786	0.767	0.741

## Discussion

We have shown that a mediator plays an imperative role in the power of a study. Without accounting for the magnitude of *b* path and the variability in the residual error of the mediator in the sample size calculation, the power of a study can be much smaller than expected. This means a larger sample size is required to detect an effect size of $a b + c^{\prime }$ for the required power. To our knowledge, this finding has not been reported in the literature and hence not being known by researchers, especially those who design for clinical trials. Our investigation confirms that one can compute the required sample size in the usual way but with a larger standard error to account for *b* and $\sigma ^2_m$. More specifically, the considered effect size shall be adjusted following Eq. (4). The role of the intervention-mediator path is trivial in the sample size/power calculation.

As noted by the reviewers, alternative way can consider the variance inflation factor (VIF) for the sample size calculation, in a similar way to the sample size calculation of cluster-randomized studies [18]. Specifically, the VIF in our context here is

$$\begin{aligned} VIF=1+ \frac{ b^2 \sigma ^2_m }{ b^2 \sigma ^2_m + \sigma ^2_y} , \end{aligned}$$

where the second term describes the proportion of a measure’s total variance that is due to the mediator-outcome path. The first step is to compute the sample size for a study as usual, assuming the absence of a mediator. The required sample size for the study that considers the presence of a mediation can then be obtained by multiplying the VIF with the sample size obtained from the former step. Furthermore, one may proceed with sensitivity analysis by considering a range of VIF when there is lack of information about the individual parameters, $b, \sigma ^2_m$ and $\sigma ^2_y$, at the design stage.

Here, we consider a simple setting with a single outcome and a single mediator, without accounting for the presence of measurement errors in both the outcome and mediator. In clinical practice, mediators are likely to be measured with an error but not the outcome. The presence of measurement error will increase the variability in a mediator. Non-differential measurement error may be captured in $\sigma ^2_m$ as each observation is assumed to have the same likelihood of being measured incorrectly, while accounting for a differential measurement error might not be straight forward, depending on the modeling assumption of the underlying mechanism. For this reason, we suggest to use historical information with care when planning the sample size of prospective trials. One may conduct a meta-analysis on $b, \sigma ^2_m$ and $\sigma ^2_y$ to inform the range of VIF and evaluate the required sample size of prospective trials accordingly. Future research may investigate the utility of historical information on the mediator alongside the idea of sample size re-estimation.

In scenarios where there is more than one mediators in the mechanism of action of intervention, one may extend the sample size calculation approach to account for the variability in the extra mediators. For example, one may follow the parallel or sequential mediator model in [13] and compute the total variability and the standardized treatment effect accordingly for the sample size calculation. In the scenarios where there are multiple mechanism of actions, we propose to proceed in a similar way to the scenarios where there are multiple outcome, e.g., consider the largest required sample sizes after computing the required sample sizes for each mediator model with or without adjustment for multiplicity.

Another limitation of our work is related to the interaction between the mediator and randomization in model (2). We assume such interaction is trivial in our investigation. In theory, one can introduce an extra term to model (2) and derive the total variability in the outcome accordingly, for computing the required sample size. In this case, it will require the knowledge of the coefficient of the interaction term at the design stage of a study. Alternatively, one may start with the required sample size as in our presentation and explore the sensitivity of the power to the presence of an interaction term, e.g., by a simulation study. Adjustment to the sample size can then be made accordingly.

In the literature of mediation analysis, it has been shown that in the absence of a direct effect, the power of testing *ab* is higher than the power of testing the total effect, i.e., *c* in model (3). For example, some authors [13] have identified scenarios when this observation holds and when it does not. Some researchers argue that this is because *a* and *b* are two coefficients and *ab* is a product; the characteristics of the product of two coefficients are not the same as that of the usual coefficient [15]. Here, we did not test *ab* using the commonly considered approaches as the assumption of having a trivial direct effect at the design stage of a clinical study is unrealistic; the model of the primary analysis rarely includes a mediator as one covariate. Whether there is a direct effect or not, the required sample size calculation is still depending on the magnitude of *b* and $\sigma ^2_m$ and one should account for these in their clinical study.

## Conclusion

Mediation analysis is often implemented as secondary analysis in clinical studies that evaluates mechanism of action of interventions. The sample size calculation for studies with efficacy and mechanism evaluation should account for the mediator-outcome association or risk the power to detect the total effect/intention-to-treat effect being lower than planned.

## Data Availability

Code to simulate data and reproduce the results in this paper can be found in the supplemental materials.

## References

[1] Dunn G, Emsley R, Liu H, Landau S, Green J, White I, et al. Evaluation and validation of social and psychological markers in randomised trials of complex interventions in mental health: a methodological research programme. Health Technol Assess (Winchester, England). 2015;19(93):1–115. 10.3310/hta19930 PMC4781463 26560448

[2] Insel TR. The NIMH experimental medicine initiative. World Psychiatr. 2015;14(2):151. 10.1002/wps.20227 PMC4471962 26043323

[3] Insel TR, Gogtay N. National Institute of Mental Health clinical trials: new opportunities, new expectations. JAMA Psychiatr. 2014;71(7):745–6. 10.1001/jamapsychiatry.2014.426 24806613

[4] Judd CM, Kenny DA. Process analysis: estimating mediation in treatment evaluations. Eval Rev. 1981;5(5):602–19. 10.1177/0193841X8100500502

[5] MacKinnon DP, Dwyer JH. Estimating mediated effects in prevention studies. Eval Rev. 1993;17(2):144–58. 10.1177/0193841X9301700202

[6] MacKinnon DP. Introduction to Statistical Mediation Analysis. New York: Taylor & Francis Group; 2008.

[7] Hayes AF. Introduction to mediation, moderation, and conditional process analysis: a regression-based approach. London: Guilford Press; 2013.

[8] VanderWeele T, Vansteelandt S. Mediation analysis with multiple mediators. Epidemiol Methods. 2014;2(1):95–115. 25580377 10.1515/em-2012-0010 PMC4287269

[9] VanderWeele TJ. Explanation in Causal Inference: Methods for Mediation and Interaction. New York: Oxford University Press; 2015.

[10] Cole DA, Maxwell SE. Testing mediational models with longitudinal data: questions and tips in the use of structural equation modeling. J Abnorm Psychol. 2003;112(4):558. 14674869 10.1037/0021-843X.112.4.558

[11] MacKinnon DP, Lockwood CM, Williams J. Confidence limits for the indirect effect: distribution of the product and resampling methods. Multivar Behav Res. 2004;39(1):99–128. 10.1207/s15327906mbr3901_4 PMC2821115 20157642

[12] Shrout PE, Bolger N. Mediation in experimental and nonexperimental studies: new procedures and recommendations. Psychol Methods. 2002;7(4):422. 12530702 10.1037/1082-989X.7.4.422

[13] O’Rourke HP, MacKinnon DP. When the test of mediation is more powerful than the test of the total effect. Behav Res Methods. 2015;47:424–42. 24903690 10.3758/s13428-014-0481-z PMC4258193

[14] Fritz MS, MacKinnon DP. Required sample size to detect the mediated effect. Psychol Sci. 2007;18(3):233–9. 17444920 10.1111/j.1467-9280.2007.01882.x PMC2843527

[15] Kenny DA, Judd CM. Power anomalies in testing mediation. Psychol Sci. 2014;25(2):334–9. 24311476 10.1177/0956797613502676

[16] Loeys T, Moerkerke B, Vansteelandt S. A cautionary note on the power of the test for the indirect effect in mediation analysis. Front Psychol. 2015;5:1549. 25628585 10.3389/fpsyg.2014.01549 PMC4290592

[17] Lee H, Cashin AG, Lamb SE, Hopewell S, Vansteelandt S, VanderWeele TJ, et al. A guideline for reporting mediation analyses of randomized trials and observational studies: the AGReMA statement. Jama. 2021;326(11):1045–56. 34546296 10.1001/jama.2021.14075 PMC8974292

[18] Donner A, Klar N. Design and analysis of cluster randomization trials in health research. London: Arnold; 2000.

